# Care-seeking behaviour and treatment practices for malaria in children under 5 years in Mozambique: a secondary analysis of 2011 DHS and 2015 IMASIDA datasets

**DOI:** 10.1186/s12936-019-2751-9

**Published:** 2019-04-02

**Authors:** Annette Cassy, Abuchahama Saifodine, Baltazar Candrinho, Maria do Rosário Martins, Saraiva da Cunha, Filomena Martins Pereira, Eduardo Samo Gudo

**Affiliations:** 1 Program of Endemic Diseases of Large Impact, Instituto Nacional de Saúde, Maputo, Mozambique; 2 Global Health and Tropical Medicine, Instituto de Higiene e Medicina Tropical, Universidade Nova de Lisboa, Lisbon, Portugal; 3 President’s Malaria Initiative, USAID, Maputo, Mozambique; 4 National Malaria Control Program, National Directorate of Public Health, Ministry of Health, Maputo, Mozambique; 5 Faculty of Medicine, University of Coimbra, Coimbra, Portugal

**Keywords:** Malaria, Care-seeking behavior, Treatment, Children under 5 years of age, Mozambique

## Abstract

**Background:**

In Mozambique, the prevalence of malaria in children under 5 years of age is among the highest in the world, but limited data exist on determinants of care-seeking behaviour for malaria. This study aimed at determining the trends and factors associated with care-seeking behaviour for fever among children under 5 years of age and to assess the treatment practices for malaria.

**Methods:**

Secondary data analysis of two cross-sectional studies. Descriptive statistics were used to summarize socio-economic and demographic characteristics of participants, using data from the 2011 Demographic and Health Survey and 2015 Indicators of Immunization, Malaria and HIV/AIDS Survey. Complex sampling logistic regression model was used to identify factors associated with care-seeking behaviour, with estimated adjusted odds ratio and respective 95% confidence intervals, only for 2015 IMASIDA data.

**Results:**

A total of 10,452 and 5168 children under 5 years of age were enrolled in the 2011 DHS and 2015 IMASIDA, respectively. Care-seeking for fever in public and private sectors remained stable during this period (62.6%; 835/1432 in 2011 and 63.7%; 974/1529 in 2015). The main place where care was sought in both surveys was public hospitals (86.2%; 773/897 in 2011 and 86.7%; 844/974 in 2015). Prescription of anti-malarial drugs increased from 42.9% (385/897) in 2011 to 53.8% (524/974) in 2015. Artemether–lumefantrine was the most used anti-malarial drug for febrile children in both surveys and its use increased from 59.0% (219/373) in 2011 to 89.3% (457/512) in 2015. Data from 2015 elucidated that care-seeking was more common in children whose mothers had a secondary level of education (AOR = 2.27 [95% CI 1.15–4.49]) and among those in poorer quintile (AOR = 1.46 [95% CI 0.83–1.90]). Mothers with higher education level (AOR = 0.16 [95% CI 0.34–0.78]) were less likely to seek out care. People from Manica (AOR = 2.49 [1.03–6.01]), Sofala ([AOR = 2.91 [1.03–8.24]), Inhambane (AOR = 3.95 [1.25–12.45]), Gaza (AOR = 3.25 [1.22–8.65]) and Maputo Province (AOR = 2.65 [1.10–6.41]) were more likely to seek care than people from Maputo City.

**Conclusion:**

Data from this study showed that care-seeking in Mozambique remained suboptimal. Interventions to raise the awareness for early care-seeking during episodes of fever should be urgently reinforced and intensified.

## Background

Mozambique is ranked among the countries with high burden of malaria [1] and in 2016, accounted for 4% of all malaria cases and 4% of all malaria deaths worldwide [1]. The disease is the leading cause of morbidity and mortality in children under the age of 5 in the country [2]. In recent years, the number of reported cases of malaria in Mozambique in the public health services has increased and its prevalence in children under 5 years of age has remained stable at 38% in 2011 and 40% in 2015 [3].

Early diagnosis and prompt and correct treatment are essential for a favourable malaria outcome, reducing its morbidity and mortality [4, 5]. Thus, care-seeking behaviour for malaria remains a cornerstone for malaria control programs [6, 7].

There is limited data on malaria care-seeking in Mozambique and there are no published reports describing factors associated with malaria care-seeking behaviour in Mozambique. There is an urgent need to determine the patterns and factors associated with care-seeking behaviour for malaria, as such knowledge is crucial for designing strategies aiming to improve malaria diagnosis and treatment [8–12]. In this context, this study was conducted with the following objectives: (i) analyse differences of care-seeking behaviour for fever in children under 5 years of age, using the 2011 Demographic and Health Survey (DHS) and 2015 Immunization, AIDS and Malaria Indicators Survey (IMASIDA) data and (ii) describe the factors associated with care-seeking behaviour and the treatment practices among children under 5 years of age, only using the 2015 IMASIDA data.

## Methods

### Study design and data source

This is a quantitative, observational study that analysed two national, cross-sectional studies in which data were collected in two periods of time: 2011 and 2015. A secondary data analysis using the 2011 DHS data and the 2015 IMASIDA data was conducted, to describe socio-economic, demographic characteristics and treatment practices. The 2015 IMASIDA data was also used to identify factors associated with care-seeking behaviour. It was decided not to use the 2011 DHS data to identify the predictors of the care-seeking, because the data was collected long time ago and so that the situation may have changed and so it does not make much sense to identify these factors.

Both surveys used nationally representative samples. The 2011 DHS included 13,964 households distributed over 611 census enumeration areas (EAs) while 2015 IMASIDA included 7169 households distributed over 307 EAs. The response rate was 98.9% and 98% in 2011 DHS and 2015 IMASIDA, respectively. Methods for both surveys have been previously described [3, 13].

### Setting

Both surveys were conducted in Mozambique. The country is located in the east coast of southern Africa and is divided in 11 provinces. Mozambique has a surface of approximately 799.380 km^2^ [14] and a population of 28.861.863 inhabitants [15]. The climate in Mozambique is tropical. The rainy season spans from October to March and the dry season occurs in the rest of the year [14]. There is year-round transmission of malaria with seasonal peaks during the rainy season. Data collection for 2011 DHS took place from June to November 2011 and for 2015 IMASIDA, from June to September 2015.

### Eligibility criteria

This analysis used data from children aged from 0 to 59 months whose mothers or guardians were interviewed and provided information on the fever in the 2 weeks prior to the surveys.

### Measures

The main outcome of this study is care-seeking behaviour of mothers/guardians of children under 5 years with history of fever in the 2 weeks prior to the survey. Potential covariates were identified for inclusion in a predictive model using literature review for “care-seeking” and “treatment-seeking” for fever and malaria. A total of 11 socioeconomic and demographic covariates previously shown to be associated with care-seeking [10, 16–24] were retrieved from 2015 IMASIDA dataset. The covariates included child’s age, sex, place of residence (urban or rural), geographic region (provinces), religion (Catholic, Muslim, Protestant or other), household wealth quintile, mother’s level of education, age and marital status (single, married/living with partner, divorced/separated or widowed), child’s use of a bed net and whether the dwelling had been sprayed with insecticide within the last 12 months or not.

As there was no variable in the database that grouped all anti-malarials to compare, a variable group was created to group them together. Marital status was originally divided into six categories (single, married, living with partner, separated, divorced, widowed) and was decided to group in four categories (single, married/living with partner, divorced/separated and widowed). Religion was divided into eight categories and was regrouped into five categories including: the three most practiced religions in Mozambique (1) Catholic, (2) Islamic and (3) Protestant [25], the (4) category combined the remaining religions, and the (5) category as the non-religious.

### Statistical analysis

Data from 2011 DHS and 2015 IMASIDA were analysed using the same statistical methods. To prepare the data for analysis, the children (KR) and individual members (PR) datasets were merged based on the unique identifier number (b16) for each survey, because the information about fever and care-seeking was available in KR file and information at a household level (use of bed net, indoor spraying) was available in the PR file.

Special (*svy)* survey commands were used to account for the complex multilevel survey design. Data were weighted to account for the differential selection probabilities at the EA, household, and individual levels so that any results with the regional weight factored into it would be representative at the national and regional level. Only weighted survey data are presented in this manuscript. Descriptive statistics were used to summarize socio-economic and demographic characteristics of participants, and comparison of care-seeking behaviours between categorical variables were assessed using Pearson Chi square test of independence. Complex sampling logistic regression model was used to identify factors associated with care-seeking behaviour, with estimated adjusted odds ratio (AOR) and respective 95% confidence intervals (CI), for 2015 IMASIDA data. All statistical analyses were performed using Stata, version 15 (Stata Corporation, College Station, Texas).

## Results

### Socio-economic and demographic characteristics of children with history of fever

As shown in Table 1, a total of 10,452 children under 5 years of age in 2011 DHS and 5168 children in 2015 IMASIDA were enrolled. The proportion of children with a history of fever doubled from 13.7% (1432/10,452) [95% CI 12.6–14.9], in 2011 to 29.6% (1529/5128) [95% CI 27.2–32.1], in 2015.

**Table 1:** Socio-economic and demographic characteristics of children under 5 years with history of fever—2011 DHS and 2015 IMASIDA

Characteristics	2011 DHS (n = 10,452)				2015 IMASIDA (n = 5168)			
	n	Fever (%)	Lower confidence	Upper confidence	n	Fever (%)	Lower confidence	Upper confidence
Child’s age in months								
< 6	1176	8.3	6.5	10.5	532	19.0	15.0	23.8
6–11	1259	16.8	14.1	199	568	32.0	27.5	36.9
12–23	2285	17.2	15.0	19.7	1116	35.3	30.9	40.0
24–35	1945	15.3	13.4	17.4	1003	32.4	28.7	36.4
36–47	1989	11.9	10.2	13.8	1019	28.9	25.0	33.0
48–59	1771	10.9	8.9	13.2	905	24.8	21.0	29.0
Missing data	27	0.3%			25	0.5%		
Sex of child								
Male	5257	14.0	12.6	15.4	2563	30.1	27.3	33.0
Female	5195	13.4	12.0	15.0	2605	29.1	26.1	32.3
Place of residence								
Urban	2840	13.3	11.3	15.5	1330	23.0	20.3	25.8
Rural	7612	13.9	12.6	15.3	3838	31.9	28.8	35.1
Region								
Northern								
Niassa	641	11.7	9.0	14.9	351	30.3	23.3	38.3
Cabo Delgado	885	15.3	12.6	18.4	466	21.8	15.6	29.7
Nampula	1581	13.3	10.4	16.2	1154	39.8	33.4	46.5
Central								
Zambézia	2188	16.6	13.1	20.7	722	52.0	46.1	57.8
Tete	1356	12.9	10.2	16.1	448	14.5	12.0	17.4
Manica	766	12.9	10.2	16.1	429	16.8	12.7	21.8
Sofala	1036	16.5	13.5	19.9	522	21.0	17.4	25.2
Southern								
Inhambane	557	8.9	6.5	12.1	284	18.2	12.1	26.4
Gaza	541	11.1	8.8	13.9	409	27.6	23.4	33.2
Maputo Province	554	10.5	8.2	13.3	209	15.4	11.2	20.8
Maputo City	348	11.0	8.5	14.1	175	24.6	18.8	31.4
Wealth index								
Poorest	2478	14.3	12.1	16.9	1212	32.8	28.8	37.1
Poorer	2278	14.9	12.8	17.2	1177	33.6	28.5	39.1
Middle	2096	13.5	11.8	15.5	1072	30.1	26.0	34.5
Richer	2069	12.7	10.6	15.1	956	26.9	23.3	30.9
Richest	1530	12.6	10.7	14.9	751	20.7	17.9	23.7
Total	10,452	13.7	12.6	14.9	5168	29.6	27.2	32.1

Fever was less common in children aged 0–6 months as compared to other age groups in both surveys, (8.3% [95% CI 6.5–10.5] in 2011 and 19.0% [95% CI 15.0–23.8] in 2015). In 2015, fever was more common in rural areas (31.9% [95% CI 28.8–35.1]) than in urban areas (20.3 [95% CI 20.3–25.8]), but this difference was not observed in 2011 (13.9% [95% CI 12.6–15.3] in rural areas and 13.3% [95% CI 11.3–15.5] in urban areas). Missing data comprised less than 1%.

### Care-seeking behaviour

Care was sought for 62.6% (835/1432; [95% CI 59.0–66.1]), of the children with a history of fever in the 2 weeks prior to the survey in 2011 and 63.7% (974/1529; [95% CI 59.3–67.8]), in 2015. Table 2 shows the demographic characteristics of children with fever for whom care was sought. In both 2011 DHS and 2015 IMASIDA, care-seeking had no significant difference between the age groups or gender.

**Table 2:** Socio-economic and demographic characteristics of children under 5 years for whom care was sought—2011 DHS and 2015 IMASIDA

Characteristics	2011 DHS (n = 1432)				2015 IMASIDA (n = 1529)			
	n	Sought care (%)	Lower confidence	Upper confidence	n	Sought care (%)	Lower confidence	Upper confidence
Child’s age in months								
< 6	97	63.9	52.7	73.7	101	67.5	56.1	77.1
6–11	212	71.7	63.2	78.9	182	67.1	58.3	74.8
12–23	394	61.4	54.9	67.4	394	63.4	57.1	69.2
24–35	298	59.5	52.5	66.2	325	65.1	57.4	72.0
36–47	237	62.2	53.8	70.0	294	62.0	53.9	69.5
48–59	193	60.2	50.1	69.5	224	59.4	50.2	68.0
Missing data	1	0.1%			9	0.6%		
Sex of child								
Male	734	61.3	56.6	65.9	771	62.6	56.6	68.3
Female	699	64.0	59.4	68.3	758	64.7	59.5	69.7
Place of residence								
Urban	377	75.0	69.8	79.7	305	74.9	68.4	80.4
Rural	1055	58.2	53.8	62.5	1224	60.9	55.6	65.9
Region								
Northern								
Niassa	75	52.2	39.4	64.8	106	51.7	42.4	60.9
Cabo Delgado	135	33.5	24.4	44.1	102	62.2	52.2	71.3
Nampula	210	86.2	75.0	92.8	459	64.2	53.9	73.4
Central								
Zambézia	362	56.6	49.4	63.5	37	58.5	48.2	68.0
Tete	175	54.4	43.5	64.8	65	63.7	41.3	81.3
Manica	99	59.3	45.3	71.9	72	68.3	59.6	75.9
Sofala	170	76.7	68.7	83.2	110	69.9	58.1	79.6
Southern								
Inhambane	49	65.8	53.2	76.5	52	77.8	64.4	87.1
Gaza	60	71.4	62.0	79.3	113	73.5	61.8	82.7
Maputo Province	58	68.9	57.7	78.2	32	76.7	63.6	86.2
Maputo City	38	69.3	54.8	80.8	43	60.2	45.8	73.0
Wealth index								
Poorest	355	56.8	49.0	64.3	398	53.9	46.0	61.7
Poorer	338	49.6	41.9	57.3	395	63.7	54.8	71.8
Middle	284	66.4	60.2	72.0	323	63.3	56.3	69.7
Richer	262	76.2	70.6	81.0	257	73.8	67.2	79.5
Richest	194	72.2	64.0	79.2	155	72.7	64.4	79.6
Total	1432	62.6	59.0	66.1	1529	63.7	59.3	67.8

In both surveys, care-seeking was higher in urban areas as compared to rural areas. In 2011, care-seeking was 75.0% [95% CI 69.8–79.7] in urban areas as compared to 58.2% [95% CI 53.8–62.5] in rural areas. Similarly, in 2015 care-seeking was 74.9% [95% CI 68.4–80.4] in urban and 60.9% [95% CI 55.6–65.9] in rural areas.

**Fig. 1:**
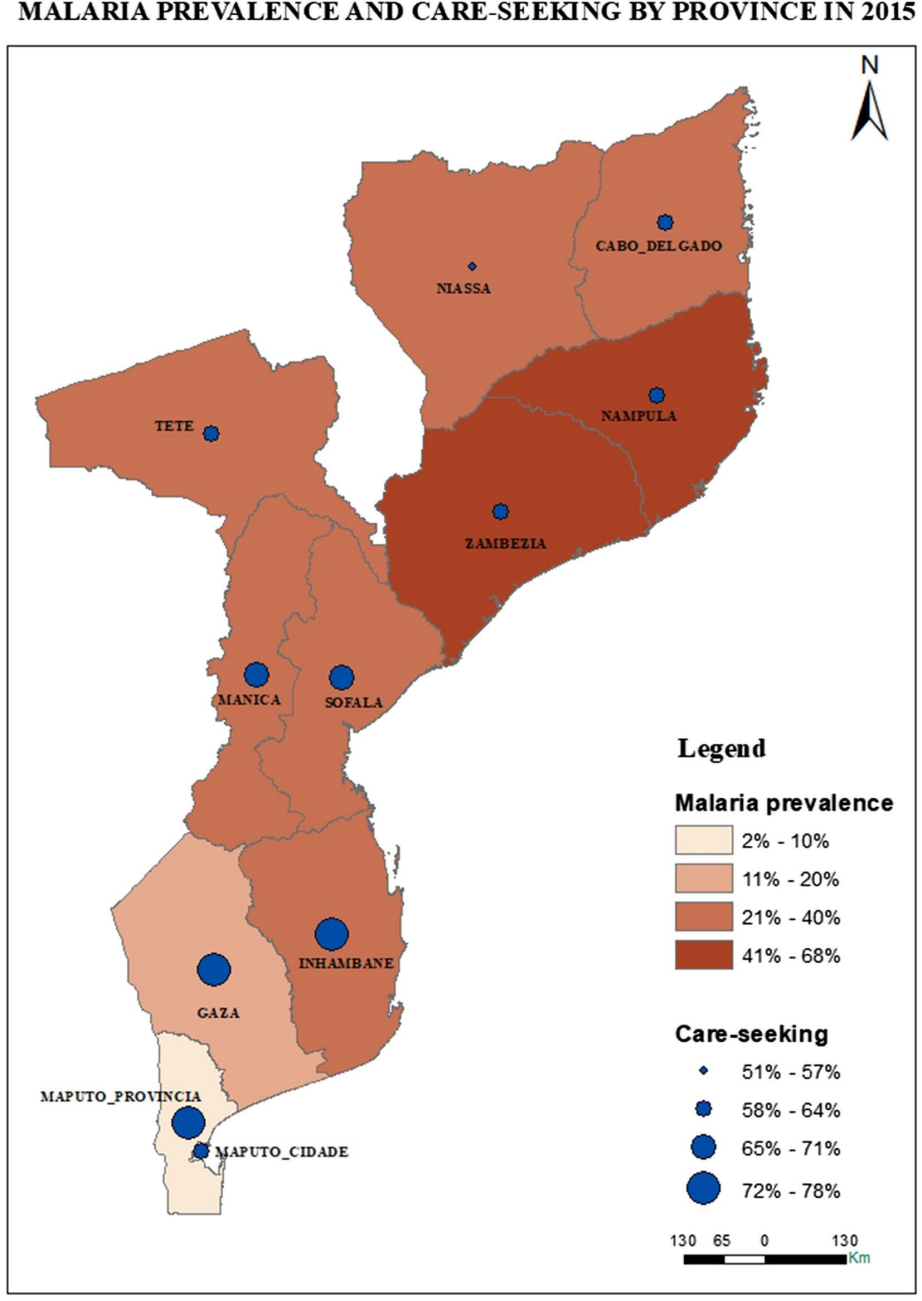
Malaria prevalence and care-seeking for fever in 2015

In 2011, Cabo Delgado province reported the lowest care-seeking (33.5%), while in 2015, Zambézia province had the lowest care-seeking (58.6%) reported. When comparing by region, the provinces situated in the southern region of the country presented the highest care-seeking behaviour, excluding Maputo City (see Fig. 1). In term of wealth index, Table 2 shows that care-seeking was lower among those in the two lower quintiles. Missing data comprised less than 1%.

Table 3 lists the places where care for fever was sought among participants of the 2011 DHS and the 2015 IMASIDA. In both surveys, care was sought mostly in public hospitals (86.2%; 773/897 in 2011 DHS and 86.7%; 844/974 in 2015 IMASIDA). Community health workers (CHW) were the second most common option for care-seeking with little difference between years 2011 (5.2%; 47/897) and 2015 (6.6%; 64/974).

**Table 3:** Place care first sought for children under five with fever—2011 DHS and 2015 IMASIDA

Place	n (%)	
	2011	2015
Public hospital	773 (86.2)	844 (86.7)
Community health worker	47 (5.2)	64 (6.6)
Other public services	10 (1.1)	12 (1.2)
Private services	10 (1.1)	23 (2.4)
Traditional healer and informal market	28 (3.1)	11 (1.1)
Informal market	21 (2.3)	12 (1.2)
Other	8 (0.9)	7 (0.8)
Total	897	974

### Malaria treatment practices

The frequency of children with fever who were treated with anti-malarial increased from 42.9% (385/897) in 2011 to 53.8% (524/974) in 2015 (Table 4). The most commonly used anti-malarial was artemether–lumefantrine and its frequency increased from 58.96% (219/373) in 2011 to 89.34% (457/512) in 2015 (Table 4). The use of other anti-malarials, such as sulfadoxine–pyrimethamine, chloroquine and quinine, reduced from 2011 (21.2%, 1.9% and 8.6%, respectively) to 2015 (5.6%, 0.3% and 0.3%, respectively).

**Table 4:** Type of anti-malarial taken for fever—2011 DHS and 2015 IMASIDA

Type of anti-malarial	n (%)	
	2011	2015
Sulfadoxine–pyrimethamine	81 (21.2)	29 (5.6)
Chloroquine	7 (1.9)	2 (0.3)
Amodiaquine	9 (2.3)	1 (0.2)
Quinine	33 (8.6)	2 (0.3)
Artemisinin combination	8 (2.0)	8 (1.6)
Artemether–lumefantrine	229 (59.5)	461 (88.0)
Other	18 (4.6)	21 (3.9)
Total	385	524

### Predictors of care-seeking behaviour for fever

Table 5 summarize the results of the multivariable analysis to identify factors associated with care-seeking behaviour for fever in 2015 IMASIDA.

**Table 5:** Logistic regression analysis of factor associated with malaria care-seeking behaviour for children under five with fever, 2015 IMASIDA

Variable	Univariable analysis			Multivariable analysis		
	OR	(95% CI)	p-value	OR	(95% CI)	p-value
Child’s age in months						
< 6	1		Reference	1		Reference
06–11	0.98	(0.55–1.75)	0.954	0.95	(0.52–1.76)	0.873
12–23	0.83	(0.51–1.38)	0.477	0.88	(0.52–1.49)	0.637
24–35	0.90	(0.52–1.56)	0.700	0.98	(0.55–1.75)	0.946
36–47	0.79	(0.46–1.36)	0.388	0.90	(0.52–1.56)	0.700
48–59	0.71	(0.41–1.21)	0.209	0.84	(0.47–1.50)	0.558
Missing data	8	0.5%				
Sex of child						
Male	1		Reference	1		Reference
Female	1.10	(0.81–1.47)	0.543	1.13	(0.83–1.53)	0.442
Type of place of residence						
Urban	1		Reference	1		Reference
Rural	0.52	(0.36–0.77)	0.001	0.65	(0.40–1.10)	0.081
Region						
North	1		Reference	1		Reference
Center	1.01	(0.67–1.53)	0.963	0.94	(0.53–1.67)	0.832
South	1.62	(1.04–2.52)	0.033	1.18	(0.61–2.31)	0.622
Wealth index						
Poorest	1		Reference	1		Reference
Poorer	1.5	(1.02–2.20)	0.04	1.47	(1.02–2.13)	0.039
Middle	1.47	(0.95–2.27)	0.081	1.38	(0.91–2.08)	0.127
Richer	2.40	(1.60–3.61)	0	1.57	(0.95–2.59)	0.075
Richest	2.27	(1.38–3.72)	0.001	0.93	(0.47–1.89)	0.850
Religion						
Catholic	1		Reference	1		Reference
Islamic	0.92	(0.62–1.37)	0.694	0.81	(0.53–1.26)	0. 354
Protestant	0.96	(0.61–1.51)	0.851	0.87	(0.49–1.54)	0.625
Others	1.43	(0.91–2.25)	0.121	1.20	(0.72–2.02)	0.478
No religion	0.59	(0.32–1.09)	0.09	0.66	(0.32–1.40)	0.279
Mother’s age						
15–24	1		Reference	1		Reference
25–34	0.67	(0.48–0.95)	0.023	0.77	(0.52–1.13)	0.183
35–49	0.52	(0.36–0.75)	0	0.65	(0.43–0.97)	0.034
Mothers’ current marital status						
Single/never married	1		Reference	1		Reference
Married/living with partner	0.39	(0.20–0.74)	0.004	0.65	(0.33–1.29)	0.215
Widowed	0.43	(0.15–1.23)	0.114	0.68	(0.19–2.41)	0.553
Divorced/separated	0.38	(0.19–0.79)	0.010	0.62	(0.29–1.34)	0.223
Mother’s highest educational level						
No education	1		Reference	1		Reference
Primary	1.6	(1.16–2.21)	0.004	1.3	(0.91–1.86)	0.142
Secondary	4.02	(2.32–6.98)	0.000	2.32	(1.18–4.56)	0.015
Higher	0.26	(0.06–1.06)	0.060	0.17	(0.04–0.76)	0.021
Missing data	9	0.6%				
Use of bed net						
No	1		Reference	1		Reference
Yes	1.15	(0.85–1.55)	0.38	1.02	(0.75–1.39)	0.892
Has dwelling been sprayed						
No	1		Reference	1		Reference
Yes	1.01	(0.67–1.53)	0.948	0.88	(0.55–1.41)	0.600
Missing data (total)				30	1.96%	

Care-seeking for fever in 2015 was significantly more likely in children whose mothers had a secondary level of education, as compared to women with no education (AOR = 2.27 [95% CI 1.15–4.49]), but women with higher education (beyond high school) were less likely to seek care (AOR = 0.16 [95% CI 0.34–0.78]). People from the poorer quintile were more likely to seek care than people from the poorest (AOR = 1.46 [95% CI 0.83–1.90]). People from rural areas were less likely to seek care than people from urban areas (AOR = 0.58 [0.34–1.00]). In relation to provinces, people from Manica (AOR = 2.49 [1.03–6.01]), Sofala ([AOR = 2.91 [1.03–8.24]), Inhambane (AOR = 3.95 [1.25–12.45]), Gaza (AOR = 3.25 [1.22–8.65]) and Maputo Province (AOR = 2.65 [1.10–6.41]) were more likely to seek care than people from Maputo City. Missing data comprised less than 2%.

## Discussion

This is the first study describing factors associated with care-seeking behaviour for fever in Mozambique. In this study the pattern of care-seeking for fever and its predictors among children under 5 years of age were investigated. The study data showed that despite the fact that the percentage of children with fever doubled from 13.7% in 2011 DHS to 27.2% in 2015 IMASIDA, care-seeking remained stable at 63% in this period. This figure is lower than the 70% defined by Mozambique’s National Malaria Control Program [26]. This finding is alarming, given that malaria is a major cause of fever in children in Mozambique and prompt care-seeking is necessary to reduce morbidity and mortality [3]. These data suggest that social and behaviour change communication directed to improving care-seeking efforts should be intensified.

The percentage of children for whom care was not sought, found in both surveys, was similar to what was reported in a study conducted in Senegal, in which 37% of children with fever did not receive any treatment or medical advice [27]. However, this figure is higher than what was reported in a study in Nigeria where care was not sought for 23% of the children [28]. The study carried out in Senegal showed that short duration of fever and rapid recovery from the disease were associated with not seeking care for fever [27]. However, in both 2011 DHS and 2015 IMASIDA, the duration of fever and severity of the disease were not documented and for this reason, any assumption on the relationship between care-seeking and the duration and severity of the febrile illness can’t be made.

This results clearly demonstrate important differences in care-seeking for fever by geographic region. Of note, Zambézia which is the province with highest malaria prevalence in the country and the second most populous province in the country, had one of the lowest reported care-seeking behaviours for fever. This low care-seeking in Zambézia has previously been found in a study on care-seeking behaviour for any disease or wound in any age [29]. The report also showed that the satisfaction with health services in the province of Zambézia was the lowest, compared to all other provinces in the country (44.5%) [29]. These findings may indicate that patient satisfaction with health services plays an important role on care-seeking behavioural outcome. Thus, Zambézia province may need further investments, not only in malaria control interventions but also in the quality of services provided in order to improve care-seeking for fever.

Higher care-seeking rates in the southern region of Mozambique might partially be related to the fact that literacy and access to medical services in the southern region is also higher [29]. This suggests that investments in social determinants of health and health systems pillars should also be considered in order to improve care-seeking for fever. Yet, despite having the highest access to health facilities (96.4%) [29], Maputo City has low care-seeking for fever. This might be associated with self-medication.

In Mozambique care for fever was mostly sought at public hospitals. This finding is different from Zambia where most of the caretakers sought treatment of fever for their children from CHW, friends, relatives, traditional healers or spiritualists [30], and from India, where traditional healers were the first choice [22]. This preference for public services should continue to be reinforced as the standard of care for diagnostic and treatment in public health facilities is good and malaria tests and treatments are provided for free. Although the CHW were the second place where care was most sought, and it did not increase significantly from 2011 to 2015, and was less frequent than what was reported in Zambia [30] and in India [31]. The fact that the proportion of people seeking care from CHWs remained almost stable from 2011 to 2015, 5.2% and 6.6%, respectively, is a surprising and concerning finding. For instance, in 2010 the Ministry of Health (MoH) started a process of expanding and improving its CHW programme, and the number of trained CHWs increased significantly during the period of the two surveys [32]. These suggests that more work is needed to improve utilization of the services provided by CHWs.

Data from 2015 IMASIDA showed that mother’s education was positively associated with care-seeking behaviour, as has been shown in other studies [17, 23, 33]. Mothers with secondary level of education were more likely to seek care than mothers with a lower education level. This lower care-seeking behaviour for fever among mother’s with low level of education can be explained by their lower awareness about etiology, prevention, diagnostics, treatment and complications of malaria [17, 33]. Despite evidence that care-seeking has been positively associated with knowledge and awareness [17], results from this study showed that mothers with the highest level of education were less likely to seek care. It is possible that because highly educated mothers have grater health literacy they rely more on self-treatment. These results also show that the association between mother’s education and care-seeking behaviour is complex. In fact, some studies failed to find any association between mother’s education and care-seeking behaviour for fever [10].

Care-seeking behaviour for fever was also associated with place of residence. Caretakers from rural areas were less likely to seek care for febrile children than those from urban areas. Similar findings have been reported in previous studies conducted in other sub Saharan Africa countries [16, 19, 34]. This was an expected finding as access to care is known to be lower in rural areas of Mozambique as compared to urban areas and people living in rural area usually travel long distances to reach health facilities [14].

Wealth of the caregiver was also associated with care-seeking behaviour for fever, a finding similar to other settings [17, 24]. Caretakers from the poorer quintile were more likely to seek care than the caretakers from the poorest quintile. This difference can be explained by lower access to health services among caregivers from the poorest quintile, as described in other settings [35].

Despite the better access and conditions found in Maputo City, care-seeking was higher in other provinces like Manica, Sofala, Inhambane, Gaza and Maputo province than in Maputo City, which might be not only associated with the self-medication mentioned before but also to the fact that the interventions to raise awareness for early care-seeking are implemented by the CHW and Maputo City is the only province in Mozambique without CHW Program [32]. Care-seeking behaviour was not influenced by child’s age or sex, a finding similar to other studies carried out in Ethiopia and Zambia [10, 30].

This study had two important limitations. First, the data related to fever and treatment practices by caretakers was self-reported. It is possible that some participants have had difficulties in recalling all relevant details or may have been influenced by social-desirability bias. However, given that only episodes of fever in the 2 weeks prior to the surveys were considered, this may have contributed to minimize the recall bias. Secondly, although both surveys used nationally and regionally representative samples, their sample sizes were different.

## Conclusion

This study showed that care-seeking for fever in children under 5 in Mozambique remained sub optimal from 2011 to 2015. This low care-seeking placed febrile children with malaria infection at serious risks of progression to severe malaria disease and death due to lack of or delayed treatment. This study highlights that interventions to raise awareness for early care-seeking during episodes of fever, along with interventions to increase community awareness about malaria treatment particularly in Zambézia Province, should be urgently reinforced and intensified in order to save lives and control the epidemic.

## Abbreviations

AIDS: Acquired Immunodeficiency Syndrome
AOR: Adjusted odds ratio
CI: Confidence intervals
CHW: Community Health Workers
DHS: Demographic and Health Survey
EA: Enumeration Area
HIV: Human Immunodeficiency Virus
IMASIDA: Indicators of Immunization, Malaria and HIV/AIDS Survey (Inquérito de Indicadores de Imunização, Malária e HIV/SIDA in portuguese)
MoH: Ministry of Health
